# Supplementary material for: Encapsulation of Hydrophobic Porphyrins into Biocompatible Nanoparticles: An Easy Way to Benefit of Their Two-Photon Phototherapeutic Effect without Hydrophilic Functionalization
Source: Cancers (Basel). 2022 May 10;14(10):2358. doi: 10.3390/cancers14102358 (PMC9139875; doi:10.3390/cancers14102358)
Supplement: Supplementary file 1 [file cancers-14-02358-s001.zip › cancers-1703973-supplementary.pdf]

Encapsulation of Hydrophobic Porphyrins into Biocompatible Nanoparticles: An Easy Way to Benefit of Their Two-Photon Phototherapeutic Effect without Hydrophilic Functionalization

Limiao Shi,[1] Christophe Nguyen,[2] Morgane Daurat,[3] Nicolas Richy,[1] Corentin Gauthier,[3] Estelle Rebecq,[3] Magali Gary-Bobo,[2] Sandrine Cammas-Marion,[1,4,*] Olivier Mongin [1], Christine O. Paul-Roth,[1,*] Frédéric Paul[1,*]

[1] *Univ Rennes, INSA Rennes, ENSCR, CNRS, ISCR (Institut des Sciences Chimiques de Rennes) – UMR 6226, F-35000 Rennes, France. E-mail: christine.paul@univ-rennes1.fr or christine.paul@insa-rennes.fr, sandrine.marion.1@ensc-rennes.fr*

[2] *IBMM, Univ Montpellier, CNRS, ENSCM, Montpellier, France. E-mail: magali.gary-bobo@inserm.fr.*

[3] *NanoMedSyn, 15 avenue Charles Flahault, 34090 Montpellier.*

[4] *INSERM, INRAE, Univ Rennes, Institut NUMECAN (Nutrition Metabolisms and Cancer) UMR_A 1341, UMR_S 1241, F-35000 Rennes, France*

Including:

1. Full synthesis of the MLABe monomer p. S2
2. Two-photon brightness and oxygen-photosensitization of **1a-b** and **2a-b** in organic media p. S2
3. Emission properties of **1b** and **2b** in THF/water [90:10] mixtures p. S3
4. Emission properties of NPs containing **1b** p. S3
5. Experimental evaluation of the porphyrin content of a given NP using UV-visible absorption spectroscopy p. S3
6. TEM-based NP's diameters p. S4
7. Study of NPs integrity after irradiation p. S4
8. References p. S6

1. Full synthesis of the MLABe monomer

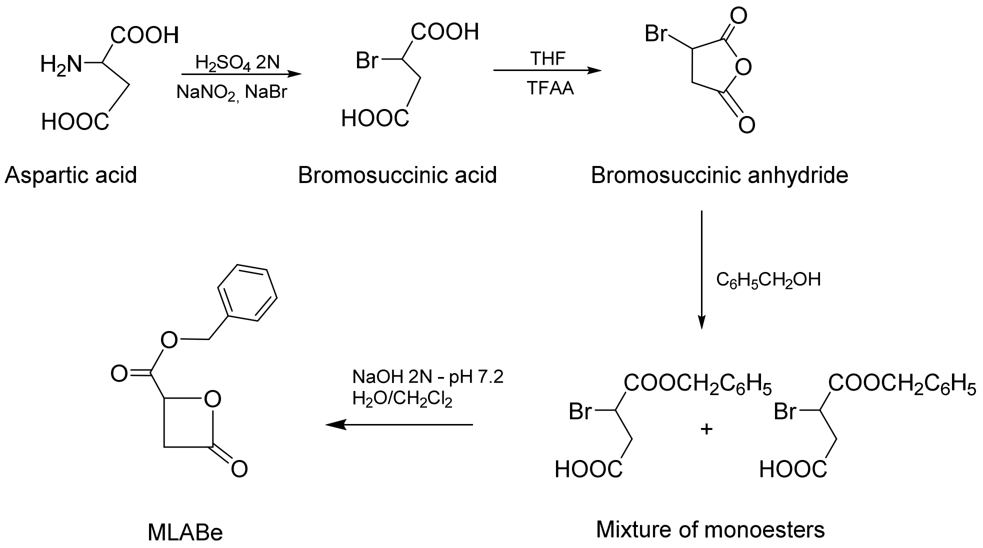

Scheme S1. *MLABe Monomer Synthesis.*

2. Two-photon brightness and oxygen-photosensitization of 1a-b and 2a-b in organic media

Table S1. 2PA and PDT data for porphyrins **1a-2a** in THF and **1b-2b** in CH_2Cl_2.[1],[2]

**Cpnd**	λ_{A2P}^{max} (nm)	$\sigma_2^{max\ a}$ (GM)	$\sigma_2^{lim}.\Phi_F$ ^{b} (GM)	Φ_Δ ^{c}	$\sigma_2^{lim}.\Phi_\Delta$ ^{d} (GM)
**1a**	790	340	68	0.60	204
**1b**	790	380	76	0.70	266
**2a**	790	810	170	0.59	478
**2b**	790	770	185	0.62	477

^{a} Intrinsic 2PA cross-sections measured in 10^{-4} M solutions by 2PEF in the femtosecond regime; a fully quadratic dependence of the fluorescence intensity on the excitation power is observed and 2PA responses are fully non-resonant. ^{b} Maximum two-photon brightness. ^{c} Singlet oxygen production quantum yield in CH_2Cl_2 determined relative to TPP in CH_2Cl_2 (Φ_F [TPP]=0.60). ^{d} Figure of merit of the two-photon excited singlet oxygen production.

3. Emission properties of 1b and 2b in THF/water [90:10] mixtures

Table S2. Photophysical data for porphyrins **1b** and **2b** in THF/H_2O [90:10] mixtures.

Porphyrin	λ_{max} (nm)	ε_{max} (M^{-1} cm^{-1})
**1b**	425	628000
**2b**	429	635000

4. Emission properties of NPs containing 1b

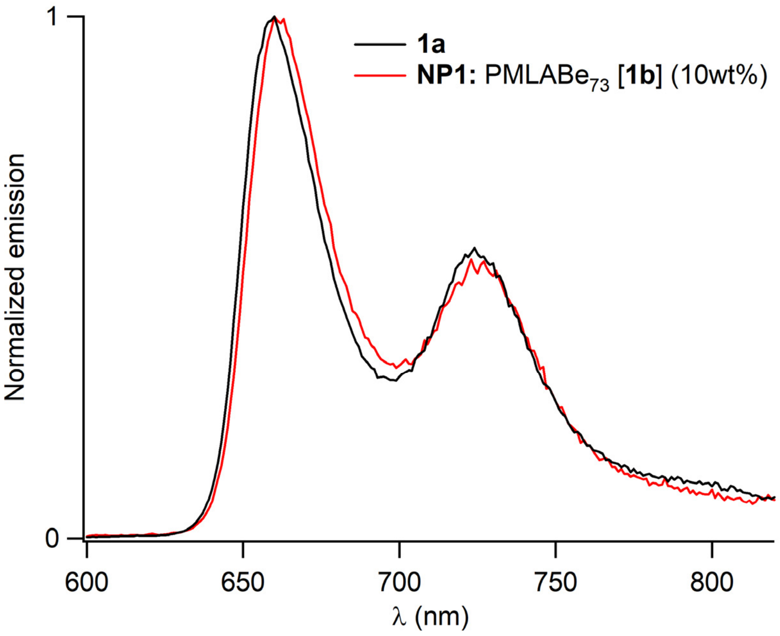

Figure S1. Comparison of the emission spectra of **NP1** incorporating 10 wt% of **1b** and the emission spectrum of their TEG-ylated analogue **1a** in water.

5. Experimental evaluation of the porphyrin content of a given NP using UV-visible absorption spectroscopy

Example of preparation of a solution in a THF/water [90:10] mixture: 0.125 mL of the polymer suspension $PMLABe_{73}$[**1b**] (10 wt%) is added to 1.125 mL of THF. The solution obtained is diluted by adding a further 2.0 mL of THF/H_2O [90:10]. The absorption spectrum is recorded and the absorbance at 425 nm is measured.

6. TEM-based NP's diameters

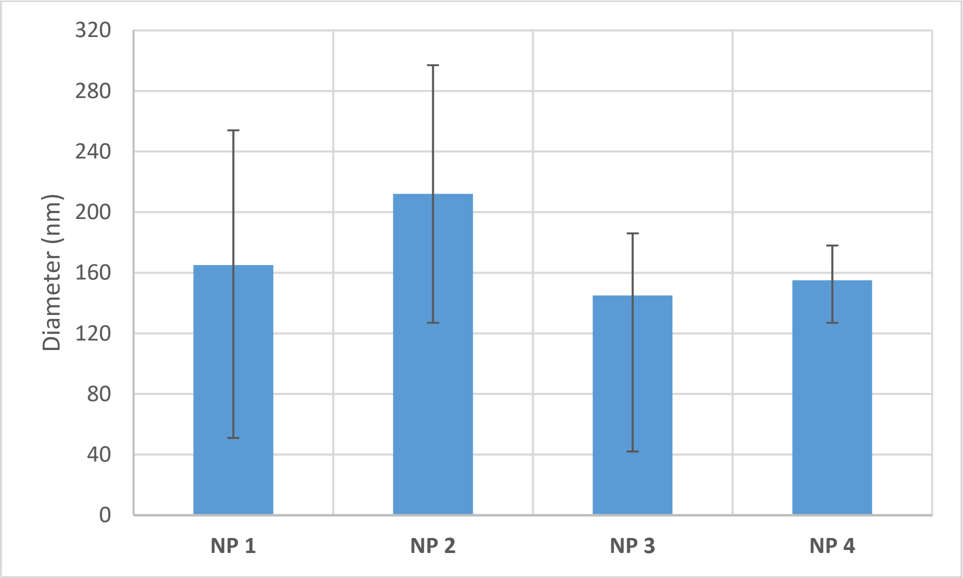

Figure S2. NP's diameters calculated for **NP1-NP4** from TEM images with experimental uncertainty.

7. Study of NPs integrity after irradiation

In order to probe any detrimental effect of irradiation on nanoparticles integrity, we have investigated the luminescence and the localization of nanoparticles (**NP1** and **NP3**) and porphyrins (**1a** and **2a**) before and after excitation at 790 nm. More precisely, cells were imaged (7% power, ×60 magnification), a picture was taken, then, cells were irradiated under 2P-PDT conditions (3 scans, 1.57 sec/scan, maximum power laser, ×10 magnification) and finally, cells were imaged again (7% power, ×60 magnification) 10, 60 or 90 min after irradiation. In figure S3, the experiment presented is shown after 60 min after irradiation. Even if irradiation seems to alter cell membrane staining, the localization and the luminescence of **NP1**, **NP3**, **1a** and **2a** does not exhibit any significant modification suggesting preservation of their structural and photochemical integrity.

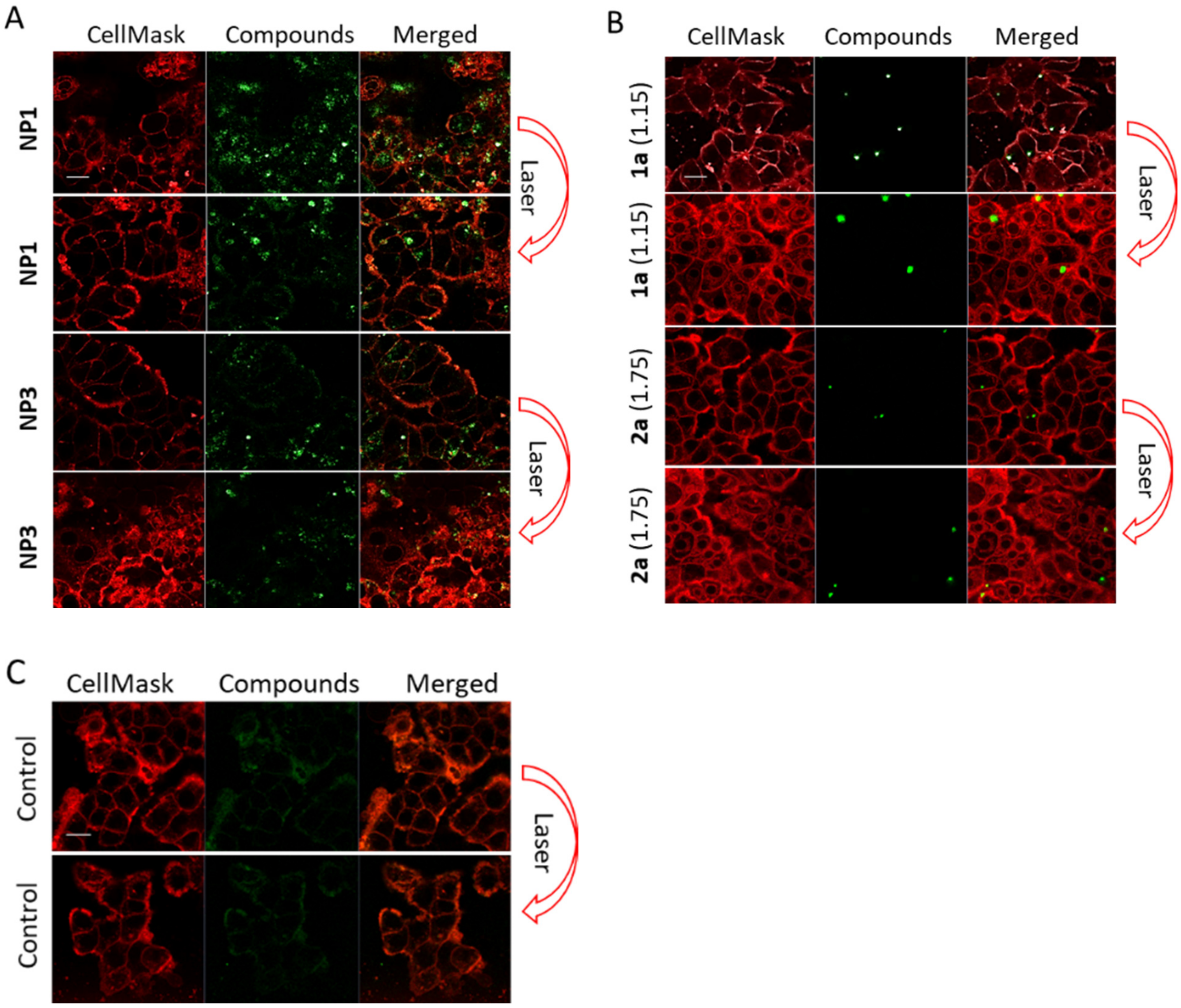

Figure S3. Porphyrin release study before and after 2PE. Fluorescence imaging of *MCF*-7 cells incubated for 20 h with **NP1, NP3** (25 µg.mL^{-1}) (**A**), with analogous TEG-ylated **1a** and **2a** (**B**) at the same porphyrin concentrations or without (**C**). Cell membranes were stained with *CellMask Orange* (in red, λ_{ex} = 561 nm). NPs and porphyrins were first imaged with a *Chameleon* femtosecond laser (in green, λ_{ex} = 790 nm,) magnification x60, then cells were excited in 2P-PDT conditions (3 scans of 1.57 s, ×10 magnification, at 100% laser power) and 60 min later, fluorescence imaging of the irradiated area was performed, ×60 magnification. Scale bar: 10 µm.

8. References

1. Yao, D.; Zhang, X.; Triadon, A.; Richy, N.; Mongin, O.; Blanchard-Desce, M.; Paul, F.; Paul-Roth, C. O., New Conjugated meso-Tetrafluorenylporphyrin-cored Derivatives as Fluorescent Two-photon Photosensitizers for Singlet Oxygen Generation. *Chem. Eur. J.* **2017,** 23, 2635-2647.
2. Shi, L.; Nguyen, C.; Daurat, M.; Dhieb, A. C.; Smirani, W.; Blanchard-Desce, M.; Gary-Bobo, M.; Mongin, O.; Paul-Roth, C.; Paul, F., Biocompatible Conjugated Fluorenylporphyrins for Two-photon Photodynamic Therapy and Fluorescence Imaging. *Chem. Commun.* **2019,** 55, 12231-12234
